# Different Associations of Coffee Consumption with the Risk of Incident Metabolic Dysfunction-Associated Steatotic Liver Disease and Advanced Liver Fibrosis

**DOI:** 10.3390/nu16010140

**Published:** 2023-12-31

**Authors:** Jun-Hyuk Lee, JooYong Park, Sang Bong Ahn

**Affiliations:** 1 Department of Family Medicine, Nowon Eulji Medical Center, Eulji University School of Medicine, Seoul 01830, Republic of Korea; swpapa@eulji.ac.kr; 2 Department of Medicine, Graduate School of Hanyang University, Seoul 04763, Republic of Korea; 3 Department of Big Data Medical Convergence, Eulji University, Seongnam-si 13135, Republic of Korea; 4 Department of Internal Medicine, Nowon Eulji Medical Center, Eulji University School of Medicine, Seoul 01830, Republic of Korea

**Keywords:** coffee, inflammation, nonalcoholic fatty liver disease, liver fibrosis, cohort, incidence

## Abstract

Although coffee has a potential hepatoprotective effect, evidence of the relationship between coffee consumption and metabolic dysfunction-associated steatotic liver disease (MASLD) remains conflicting. There is limited evidence regarding the most appropriate coffee intake to prevent advanced liver fibrosis (ALF) in patients with MASLD. We investigated the effect of coffee consumption on MASLD and ALF among 5266 participants without MASLD and 1326 with MASLD but without ALF. Participants were grouped by coffee intake: non-consumers, >0 and <1 cups/day, ≥1 and <2 cups/day, and ≥2 cups/day. Over a median follow-up of 11.6 years for MASLD and 15.7 years for ALF, coffee consumption did not significantly affect the incidence of MASLD, with 2298 new cases observed. However, a notable inverse association was found with ALF risk in patients with MASLD among those consuming coffee ≥2 cups/day (adjusted HR 0.57, 95% CI: 0.37–0.90, *p* = 0.014), especially among those consuming coffee ≥2 and <3 cups/day (adjusted HR 0.51, 95% CI: 0.30–0.89, *p* = 0.018). This suggests a potential hepatoprotective effect of coffee, especially in preventing the progression of liver fibrosis in patients with MASLD. These findings propose that coffee consumption could be a simple and effective approach to mitigate the risk of ALF in individuals with MASLD.

## 1. Introduction

Metabolic dysfunction-associated steatotic liver disease (MASLD), a new consensus-driven proposed nomenclature replacing “nonalcoholic fatty liver disease (NAFLD)” [1], affects a substantial portion of the global population and poses a growing public health concern because of its potential to progress to advanced liver fibrosis (ALF) and other severe liver complications [2]. The prevalence of liver fibrosis is estimated to be 2.8% in the overall population, and 13% of these cases are attributable to MASLD [3]. Unlike other histologic features, the fibrosis stage of the liver was a strong independent predictor for long-term mortality, liver transplantation, and liver-related events [4,5,6]. In a large cohort study with a median follow-up of 14.2 years, the risk of mortality increased progressively with the histological stages of MASLD when compared to the control group. Specifically, the risk of mortality is 1.71 times higher in individuals with simple steatosis, 2.14 times higher in those with non-fibrotic metabolic dysfunction-associated steatohepatitis, 2.44 times higher in patients with non-cirrhotic liver fibrosis, and 3.79 times higher in patients with liver cirrhosis [7]. Furthermore, among the U.S. general population, the prevalence of fibrotic metabolic dysfunction-associated steatohepatitis, a condition accompanied by significant liver fibrosis (≥F2) and an elevated NAFLD activity score ≥ 4, has increased from 6.9% in 1999–2004 to 9.2% in 2011–2016 [8]. Notably, there has been a rise in the prevalence of fibrotic metabolic dysfunction-associated steatohepatitis among individuals with obesity and diabetes, with inflammation playing a pivotal role [9,10,11]. Therefore, it is important to detect and manage the risk factors for liver fibrosis to prevent the development of ALF. Emerging evidence suggests that lifestyle modifications, including dietary interventions, play a crucial role in the management and prevention of MASLD progression [12,13,14]. Coffee, a widely consumed beverage, has been proposed to possess hepatoprotective properties. Possible hepatoprotective mechanisms associated with coffee consumption have been proposed to include: (1) suppression of genes promoting fibrosis; (2) prevention of hepatic stellate cell adhesion and activation; (3) stimulation of the Nrf2 pathway, which induces the antioxidant enzyme system; and (4) reduction of liver inflammation [15,16].

The association between coffee consumption and liver disease has been studied in various populations, with some studies reporting a reduced risk [17,18,19,20]. However, there is conflicting evidence regarding the relationship between coffee consumption and the incidence of MASLD [21,22]. Most previous studies were limited by their cross-sectional design, small sample sizes, and failure to account for potential confounding factors, such as alcohol consumption, smoking, other dietary factors, and metabolic disorders [23,24]. Additionally, there is a lack of evidence on the dose–response relationship between coffee consumption and ALF risk in individuals with MASLD, as well as the most appropriate amount of coffee consumption to reduce the incidence of ALF. Therefore, a robust analysis of the association between coffee consumption and ALF risk in individuals with MASLD could provide evidence-based recommendations for appropriate coffee intake levels to manage MASLD, potentially contributing to cost-effective public health strategies.

This study aimed to investigate the association between coffee consumption and the risk of incident ALF in patients with MASLD as well as in individuals without MASLD using data from a large community-based cohort with up to 16 years of follow-up data.

## 2. Materials and Methods

### 2.1. Study Population

We analyzed data from the Korean Genome and Epidemiology Study (KoGES)_Ansan_Ansung cohort. The KoGES_Ansan_Ansung cohort is a community-based prospective cohort study conducted by the Korea Disease Control and Prevention Agency. In the baseline survey (2001–2002), 10,030 participants aged 40–69 years living in urban (Ansan) and rural (Ansung) areas for at least six months were recruited. The participants were followed up biennially until the eighth follow-up period (2017–2018). In this longitudinal study, we utilized independent variables and confounding variables from the initial survey such as personal diet, medical history, anthropometric measurements, and blood samples. For the analysis of incident MASLD and ALF (main outcome), we employed longitudinal data, starting from the initial survey and extending through the latest follow-up. Individuals who did not have MASLD at baseline were monitored to observe the incidence of MASLD. Conversely, individuals who were diagnosed with MASLD at baseline were specifically observed for the development of ALF. Participants were evaluated biennially, during which we collected data on current prevalent diseases through questionnaires, anthropometric measurements, and laboratory tests. The data on coffee consumption was sourced exclusively from the initial survey, whereas the information regarding MASLD and ALF status was gathered both at the initial survey and during each subsequent visit of the participants.

**Figure 1:**
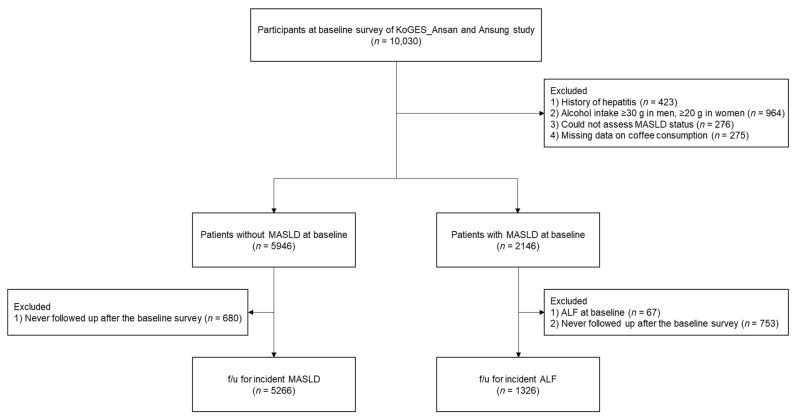
Flowchart of the study population. Abbreviations: KoGES, Korean Genome and Epidemiology Study; MASLD, metabolic dysfunction-associated steatotic liver disease; ALF, advanced liver fibrosis.

A flowchart of the study population selection is presented in Figure 1.

Of the 10,030 participants who participated in the baseline survey, we excluded the following: (1) those with a history of hepatitis (*n* = 423); (2) men whose alcohol consumption was ≥30 g/day or women whose alcohol consumption was ≥20 g/day (*n* = 964); (3) those with insufficient data to assess MASLD status (*n* = 276); and (4) those with missing data on coffee consumption (*n* = 275). Among the remaining 8092 participants with or without MASLD at baseline, we analyzed data from 5266 participants without MASLD for follow-up incident MASLD after excluding those who were not followed up after the baseline survey (*n* = 680) and data from 1326 participants with MASLD at baseline for follow-up incident ALF after excluding those with ALF (*n* = 67) and those who were not followed up after the baseline survey (*n* = 753).

The KoGES_Ansan_Ansung cohort protocol was reviewed and approved by the Institutional Review Board of the Korea Disease Control and Prevention Agency. Written informed consent was obtained from all participants. The study protocol conformed with the ethical guidelines of the 1964 Declaration of Helsinki and its subsequent amendments. This study was approved by the Institutional Review Board of the Nowon Eulji Medical Center (Institutional Review Board number: 2023-05-007).

### 2.2. Dietary Assessment

Each participant’s diet was assessed using a well-validated 103-item food frequency questionnaire [25]. Individuals were interviewed one-on-one by a well-trained dietitian, who asked about their average frequency of consumption and the amount consumed per serving of each of the 103 foods over the past year. The CAN-Pro 5.0 program, provided by the Korean Nutrition Society, includes the detailed nutrient composition of 103 food items in the food frequency questionnaire based on the standard serving size of each food. Using the program, we calculated the total energy intake (kcal/day), total fat intake (g/day), polyunsaturated fatty acid (g/day), monounsaturated fatty acid (g/day), and saturated fatty acid (g/day).

For questionnaires about coffee consumption, the response options for the coffee consumption frequency were “Almost never”, “Once a month”, “2–3 times a month”, “1–2 times a week”, “3–4 times a week”, “5–6 times a week”, “1–2 times a day”, “3–4 times a day”, and “5–6 times a day”. The response options for the average serving size of coffee were “1/2 cup”, “1 cup”, and “1.5 cups”. Examples of pictures corresponding to each response option were provided to help the respondents estimate their average serving size. Average daily coffee consumption (cups/day) was calculated by multiplying the average serving size by the average frequency of coffee consumption per day. We categorized participants into the group without coffee consumption, the group with >0 and <1 cups/day, the group with ≥1 and <2 cups/day, and the group with ≥2 cups/day. Average daily sweetened beverage consumption (cups/day) was calculated using the same method as that used to calculate average daily coffee consumption.

### 2.3. Definition of MASLD and ALF

MASLD was defined as an the NAFLD-liver fat score >−0.640 [26]. Participants without MASLD were followed up from the date of the baseline survey until the first MASLD event was ascertained, the end date of the study, or the date of the last informative contact. ALF was defined using fibrosis index-4 (FIB-4), a well-validated surrogate marker of liver fibrosis. We defined ALF as a FIB-4 score ≥ 2.67 [27]. Incident ALF was defined as new-onset ALF during the follow-up period among patients with MASLD. Participants with MASLD were followed up from the date of the baseline survey until the first ALF event was ascertained, the end date of the study, or the date of the last informative contact.

### 2.4. Measurements

The height and weight of the participants were recorded with a precision of 0.1 cm and 0.1 kg, respectively. The body mass index (BMI) was calculated based on these measurements. Systolic blood pressure and diastolic blood pressure were measured thrice, and the average of the last two measurements was used to calculate the mean blood pressure. Smoking status was classified into four categories: never a smoker, former smoker, intermittent smoker, and daily smoker. For alcohol consumption, questionnaires for the drinking frequency, the type of glasses, and the number of glasses per time according to the specific types of drink (makgeolli, beer, cheongju, wine, soju, and whisky) were obtained. The response options for drinking frequency were “Once a month”, “2–3 times a month”, “Once a week”, “2–3 times a week”, “4–6 times a week”, and “Every day”. The response options for the type of glasses for each type of drink consist of “soju glass”, “beer glass”, and “whisky glass”. Participants reported the number of glasses they consumed per occasion for each type of drink in the questionnaire. The amount of alcohol intake (g/time) was calculated by summing the alcohol content from the consumption of various beverages, including makgeolli, beer, cheongju, wine, soju, and whisky. For this calculation, we utilized the ethanol specific gravity of 0.7893. The respective alcohol contents for each beverage are as follows: makgeolli 6%, beer 4.5%, cheongju 15%, wine 13%, soju 22%, and whisky 40%. Finally, the amount of alcohol intake (g/day) was calculated by multiplying the frequency of alcohol intake (time/day) and the amount of alcohol intake per time (g/time). After excluding men whose alcohol consumption was ≥30 g/day and women whose alcohol consumption was ≥20 g/day, we classified participants into non-drinkers and current drinkers. Physical activity was measured using the International Physical Activity Questionnaire—long form [28], and the results were reported as the metabolic equivalent of task hours per day (MET-hr/day). The participants were grouped into three categories based on their physical activity levels: low (<7.5 MET-hr/day), moderate (7.5–30 MET-hr/day), and high (>30 MET-hr/day). The monthly household income was categorized into three groups: <KRWKRW100–200 million, and >KRW200 million. Educational levels were classified into three groups: elementary school or middle school, high school, and college or university.

Blood samples were collected from each participant after at least an 8 h fast, and multiple biomarkers were analyzed, including whole blood platelet count, fasting plasma glucose (FPG), serum levels of aspartate aminotransferase (AST), alanine aminotransferase (ALT), gamma-glutamyl transpeptidase (r-GTP), total bilirubin, albumin, glycosylated hemoglobin (HbA1c), total cholesterol, triglycerides, high-density lipoprotein (HDL) cholesterol, and C-reactive protein. Serum high-sensitivity C-reactive protein (hsCRP) levels were calculated based on the previous literature, as follows [29]:

hsCRP (mg/L)=C-reactive protein (mg/dL) ÷ 9.2

Serum low-density lipoprotein (LDL) cholesterol levels were calculated using the Friedewald equation if the serum triglyceride level was less than 400 mg/dL.

Diabetes mellitus (DM) was diagnosed if any of the following criteria were met: (1) FPG ≥ 126 mg/dL, (2) plasma glucose level ≥ 200 mg/dL 2 h after the 75 g oral glucose tolerance test, (3) serum HbA1c level ≥ 6.5%, (4) treatment with anti-diabetic medications, or (5) treatment with insulin therapy [30]. Hypertension (HTN) was diagnosed if any of the following criteria were met: (1) systolic blood pressure ≥ 140 mmHg, (2) diastolic blood pressure ≥ 90 mmHg, or (3) treatment with anti-hypertensive medications [31]. Dyslipidemia was diagnosed if any of the following criteria were met: (1) serum total cholesterol ≥ 240 mg/dL, (2) serum triglyceride ≥ 200 mg/dL, (3) serum HDL cholesterol < 40 mg/dL, or (4) serum LDL cholesterol ≥ 160 mg/dL [32].

### 2.5. Statistical Analysis

Continuous variables are presented as mean ± standard deviation or median (25th, 75th) based on the results of the Kolmogorov–Smirnov test, whereas categorical variables are presented as number and percentage (%). Analysis of variance was used for continuous variables and the chi-square test was used for categorical variables to compare differences among the coffee consumption groups.

Cox proportional hazard regression analysis and Kaplan–Meier curves were used to investigate the relationship between coffee consumption and incident MASLD or ALF, as well as the dose–response relationship between coffee consumption and incident MASLD or ALF. In patients without MASLD, a Cox proportional hazard spline curve was used to draw the dose–response relationship between coffee consumption and incident MASLD, whereas in patients with MASLD and without ALF, a Cox proportional hazard spline curve was used to draw the dose–response relationship between coffee consumption and incident ALF. Kaplan–Meier curves were used to compare the cumulative incidence rates of incident MASLD or ALF among the different coffee consumption groups. Univariate and multivariable Cox proportional hazard regression analyses were used to estimate the hazard ratio (HR) with a 95% confidence interval (CI) for incident MASLD or ALF in the different coffee consumption groups compared with the group without coffee consumption. The same confounding variables were adjusted in both analyses, including age, sex, BMI, physical activity, smoking status, alcohol drinking status, education level, monthly household income, total energy intake, average sweetened beverage consumption, whole blood platelet count, serum ALT, r-GTP, albumin, hsCRP levels, DM, HTN, and dyslipidemia. To identify the most appropriate amount of coffee consumption to prevent incident ALF in patients with MASLD, we performed a sensitivity analysis for incident ALF in patients with MASLD by dividing the group with ≥2 cups of coffee intake into two subgroups: those with ≥2 and <3 cups and those with ≥3 cups of coffee intake. Using a linear mixed model, we compared the longitudinal changes in FIB-4 scores in patients with and without MASLD across the different coffee consumption groups.

All statistical analyses were conducted using SAS version 9.4 (SAS Institute Inc., Cary, NC, USA) and R software (version 4.1.3; R Foundation for Statistical Computing, Vienna, Austria). Statistical significance was set at *p* < 0.05.

## 3. Results

### 3.1. Baseline Characteristics of the Study Population

Table 1 presents the baseline characteristics of individuals without MASLD as well as individuals with MASLD and without ALF, categorized according to their coffee consumption.

For individuals without MASLD, the group that did not consume coffee had the highest mean age, blood pressure, whole blood platelet count, serum LDL cholesterol levels, percentages of carbohydrate intake and protein intake to total energy intake, and polyunsaturated fatty acids intake to total energy intake. Additionally, this group had the highest median FIB-4 score. Regarding lifestyle and socioeconomic factors, they had the highest proportion of never smokers and individuals engaging in high-intensity physical activity. This group also predominantly consisted of individuals with low education levels and low monthly household incomes. Furthermore, patients with HTN were most prevalent in this group compared to all other groups. The group that consumed ≥2 cups/day of coffee had the highest mean values of whole blood platelet count, total energy intake, and percentage of fat intake to total energy intake. This group also had the highest proportion of men, daily smokers, individuals with high education levels, and individuals with high monthly household incomes. The group that consumed >0 but <1 cups/day of coffee exhibited the highest mean serum AST and ALT levels. Meanwhile, the group consuming ≥1 but <2 cups/day had the highest mean serum total bilirubin, albumin levels, and percentages of saturated fatty acids intake and monounsaturated fatty acids intake to total energy intake.

For individuals with MASLD but without ALF, the group that did not consume coffee had the highest mean age, blood pressure, percentages of carbohydrate intake and protein intake to total energy intake, median FIB-4 score, and proportion of patients with HTN. In contrast, those who consumed ≥2 cups/day of coffee showed the highest proportions of men, everyday smokers, current alcohol drinkers, and individuals with high education levels and high monthly household incomes. This group also had the highest mean values of serum ALT level, serum LDL cholesterol level, total energy, and percentage of fat intake to total energy intake. Notably, the group consuming >0 but <1 cups/day of coffee had the lowest mean serum ALT level. Meanwhile, the group consuming ≥1 but <2 cups/day of coffee had the lowest proportion of patients with HTN among all groups.

### 3.2. Association of Coffee Consumption and the Risk of Incident MASLD among People without MASLD

During the median 11.6 years of follow-up, there were 2298 (43.64%) newly developed MASLD cases among a total of 5266 participants without MASLD. According to the Cox proportional hazard spline curve, the HR for incident MASLD did not change regardless of the amount of coffee consumed (Figure S1: HR = 1.03; 95% CI: 0.98–1.09; *p* = 0.260).

**Figure 2:**
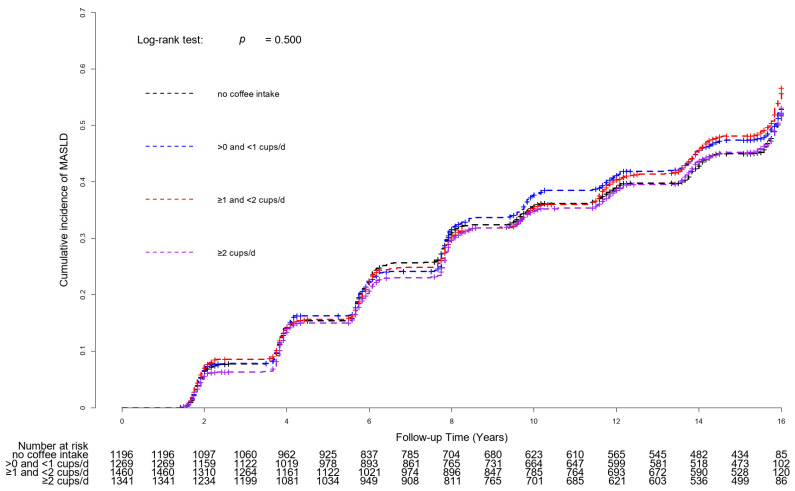
Kaplan–Meier curves with log-rank tests for cumulative incident MASLD rates based on coffee consumption. Abbreviation: MASLD, metabolic dysfunction-associated steatotic liver disease.

Figure 2 shows the Kaplan–Meier curves with log-rank tests for the cumulative incident MASLD rates based on coffee consumption. There were no significant differences in the cumulative incidence rates of MASLD between coffee consumption groups (*p* for log-rank test = 0.500).

Table 2 presents the Cox proportional hazards regression models for incident MASLD based on coffee consumption. Compared to the group without coffee consumption, the unadjusted HR (95% CI) for incident MASLD of the group with >0 and <1 cups/day, group with ≥1 and <2 cups/day, and group with ≥2 cups/day was 1.04 (0.92–1.18, *p* = 0.497), 1.07 (0.96–1.21, *p* = 0.231), and 1.00 (0.89–1.13, *p* = 0.970), respectively. In the adjusted model, the adjusted HR (95% CI) for incident MASLD in the group with >0 and <1 cups/day, group with ≥1 and <2 cups/day, and group with ≥2 cups/day were 1.04 (0.91–1.17, *p* = 0.595), 1.05 (0.93–1.19, *p* = 0.447), and 0.98 (0.86–1.18, *p* = 0.769), respectively.

### 3.3. Association of Coffee Consumption and the Risk of Incident ALF among Patients with MASLD

During the median 15.7 years of follow-up, there were 207 (15.61%) patients with newly developed ALF among the 1326 patients with MASLD. Figure S2 shows the inverse dose–response relationship between coffee consumption and HR for ALF incidence. As the frequency of coffee consumption increased, the risk of developing ALF decreased (HR = 0.79; 95% CI: 0.67–0.93; *p* = 0.006).

**Figure 3:**
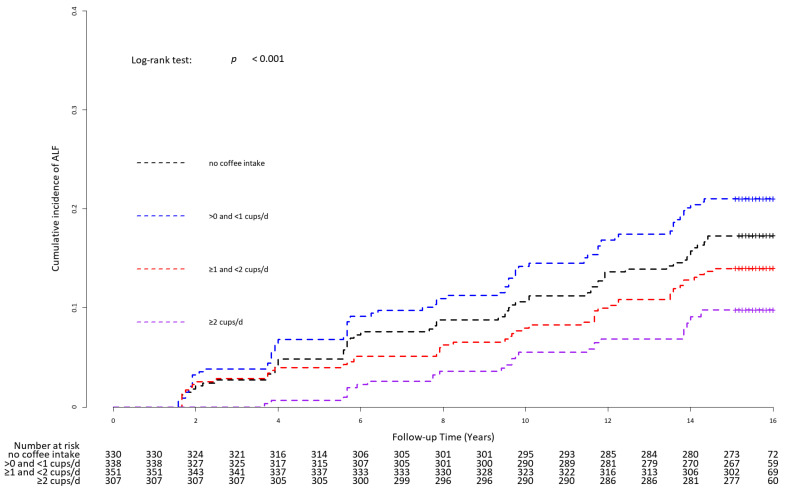
Kaplan–Meier curves with log-rank test for cumulative incidence rate of ALF for the different coffee consumption groups. Abbreviation: ALF, advanced liver fibrosis.

Figure 3 presents the Kaplan–Meier curves with the log-rank test for the cumulative incident rate of ALF for the different coffee consumption groups. The cumulative incident rate of ALF was highest in the group consuming >0 and <1 cups/day, followed by the group without coffee consumption, the group with ≥1 and <2 cups/day, and the group with ≥2 cups/day (*p* for log-rank test = 0.001).

Table 3 shows the univariate and multivariable Cox proportional hazards regression analyses for ALF incidence and the potential risk factors. The incident rates of ALF per 1000 person–years were 11.94, 14.93, 9.47, and 9.44 in the group without coffee consumption, >0 and <1 cups/day, ≥1 and <2 cups/day, and ≥2 cups/day, respectively. Compared to the group without coffee consumption, the HR (95% CI) for ALF incidence of the group with >0 and <1 cups/day, group with ≥1 and <2 cups/day, and group with ≥2 cups/day was 1.20 (0.87–1.65, *p* = 0.276), 0.76 (0.53–1.07, *p* = 0.115), and 0.64 (0.43–0.91, *p* = 0.015), respectively. A significant inverse relationship between coffee consumption and ALF incidence was observed in the adjusted model. The corresponding adjusted HR (95% CI) in the group with >0 and <1 cups/day, group with ≥1 and <2 cups/day, and group with ≥2 cups/day were 1.04 (0.73–1.49, *p* = 0.820), 0.80 (0.55–1.17, *p* = 0.248), and 0.57 (0.37–0.90, *p* = 0.014), respectively.

Table 4 shows that the inverse association between coffee consumption and ALF incidence remained significant in the group with ≥2 and <3 cups/day, but not in the group with ≥3 cups/day. The adjusted HRs (95% CI) for ALF incidence in the group with ≥2 and <3 cups/day and the group with ≥3 cups/day compared to the group without coffee consumption were 0.51 (0.30–0.89, *p* = 0.018) and 0.65 (0.38–1.12, *p* = 0.119), respectively.

### 3.4. Longitudinal Changes in FIB-4 Scores by Coffee Consumption Groups in Patients with MASLD

**Figure 4:**
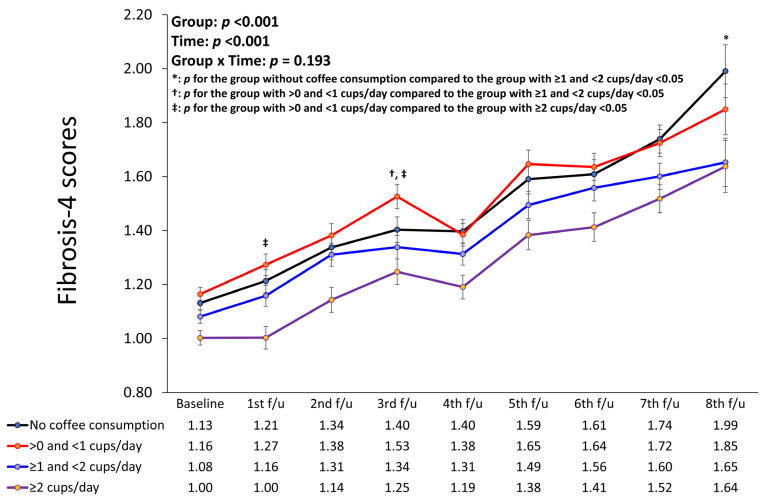
Linear mixed model showing longitudinal changes in fibrosis-4 scores of different coffee consumption groups. *: *p* for the group without coffee consumption compared to the group with ≥1 and <2 cups/day <0.05; ^†^: *p* for the group with >0 and <1 cups/day compared to the group with ≥1 and <2 cups/day <0.05; ^‡^: *p* for the group with >0 and <1 cups/day compared to the group with ≥2 cups/day <0.05.

Figure 4 presents a linear mixed model showing the longitudinal changes in the FIB-4 scores of the different coffee consumption groups after adjusting for confounding factors. The group with a coffee consumption of ≥2 cups/day had the lowest FIB-4 scores compared to the other groups throughout all periods (*p* for group < 0.001, *p* for time < 0.001, *p* for interaction of group with time = 0.193). In addition, the group without coffee consumption had the highest FIB-4 scores during the fourth, seventh, and eighth follow-up periods, whereas the group with >0 and <1 cups/day coffee consumption had the highest FIB-4 scores during the remaining periods. Although the overall interaction of groups with time was not significant, there were significant changes in FIB-4 scores from baseline to the first follow-up period between the group with >0 and <1 cups/day and the group with ≥2 cups/day. In addition, there were significant changes from baseline to the third follow-up period between the group with >0 and <1 cups/day and the group with ≥1 and <2 cups/day; between the group with >0 and <1 cups/day and the group with ≥2 cups/day; and further, from baseline to the eighth follow-up period between the group without coffee consumption and the group with ≥1 and <2 cups/day.

## 4. Discussion

We found that coffee consumption limited or ameliorated the subsequent development of ALF in patients with MASLD but had no effect on the development of MASLD in individuals previously free of MASLD. Individuals with MASLD who consumed ≥2 cups of coffee daily had a lower risk of developing ALF. The sensitivity analysis showed that this association was significant only in the group consuming ≥2 and <3 cups of coffee per day but not in the group consuming ≥3 cups per day. This suggests that the most appropriate protective effect of coffee consumption against the development of ALF in patients with MASLD may occur with a daily intake of two cups of coffee. While there was an inverse dose–response relationship between coffee consumption and the risk of incident ALF, the cumulative incidence rate of ALF was the highest in the group without coffee consumption. However, the HR (95% CI) between the group without coffee consumption and the group with >0 and <1 cups/day was not statistically significant, and the group with the highest mean FIB-4 score changed during the follow-up period. To confirm whether this is the most appropriate amount of coffee consumption to prevent the occurrence of ALF in patients with MASLD, external validation studies with larger sample sizes should be performed.

In human studies, evidence of the preventive role of coffee consumption in the development of MASLD is heterogeneous [21,22,24]. Two meta-analyses revealed that the risk of MASLD was significantly lower in patients who drank coffee compared to those who did not, with a pooled relative risk of 0.77–0.71, and the risk of liver fibrosis was also significantly decreased in MASLD patients who drank coffee regularly, with a pooled relative risk of 0.68–0.70 [21,24]. However, the main limitation of these meta-analyses is the variation in the definition of regular coffee consumption across the included studies, ranging from the consumption of more than one cup per day to more than three cups per day as the exposure definition. Consequently, these articles were unable to specify the exact number of coffee drinks. In contrast, our study was able to present a more detailed account of coffee intake. Meanwhile, a recent meta-analysis conducted by Kositamongkol et al. [22] revealed that coffee consumption was associated with a reduced risk of liver fibrosis in patients with MASLD (odds ratio = 0.67; 95% CI:0.55–0.80) and was not associated with the development of MASLD in the general population. Our results support and expand upon the evidence suggested by Kositamongkol et al. [22], as we have verified the long-term association of coffee consumption with incident ALF as well as incident MASLD by analyzing data from a community-based cohort. Given the conflicting evidence regarding the effect of coffee consumption on the development of MASLD, dietary factors related to a high-fat diet may play a significant role. Moreover, a recent cohort study using UK biobank data found that all types of coffee exhibited a protective effect against the development of chronic liver disease [33]. Over a median follow-up period of 10.7 years, coffee drinkers, compared to non-coffee drinkers, had a lower risk of incident chronic liver disease (HR of 0.79) and mortality from chronic liver disease (HR of 0.51). These significant associations were also observed in individuals who consumed decaffeinated, instant, and ground coffee. The greatest protective effect was observed with a coffee consumption of 3–4 cups/day. Conversely, a Mendelian randomization study, also using UK biobank data, found no significant association between coffee and MASLD, with an odds ratio of 0.76 and a 95% CI of 0.51–1.14 [34]. The causal relationship was determined using four-single nucleotide polymorphism (SNP) and six-SNP instruments derived from large GWAS datasets on habitual coffee consumption. To enhance the statistical power, up to 77 SNPs associated with coffee intake were also used. However, all analyses—whether using the four-SNP, six-SNP, or seventy-seven-SNP instruments—indicated non-significant trends pointing towards coffee’s protective effect against MASLD. The limited variance captured by the selected SNPs might explain the inconclusive results. Our findings align with this previous study. In our research, however, the most pronounced protective effect against the onset of ALF was noted at 2–3 cups/day, with a diminishing effect observed beyond 3 cups/day. Moreover, the reasons for the non-significant relationship between coffee consumption and incident MASLD in our study might differ from those in previous research. The discrepancies might be due to our study’s smaller sample size, genetic variations based on ethnicity, or variations in coffee preparation, such as the addition of sugar.

In an animal study, coffee prevented high-fat diet-induced MASLD in mice by modifying pathways related to liver fat oxidation, intestinal cholesterol efflux, energy metabolism, and gut permeability, while also affecting the gut microbiota [35]. Mice in the high-fat diet with coffee group showed a reduction in liver fat accumulation. This suggests that the preventive effect of coffee may be more pronounced in the presence of a high-fat diet. However, the traditional Korean diet mainly consists of carbohydrates and fiber, and Koreans tend to consume fats in an almost equal saturated fatty acid: monounsaturated fatty acid: polyunsaturated fatty acid ratio of 1:1:1, with a total fat intake below 20% of the total energy intake [36]. Similarly, in this study, individuals without MASLD consumed fats mostly as polyunsaturated fatty acids (14.9 ± 8.0 g/day), followed by monounsaturated fatty acids (11.2 ± 6.6 g/day) and saturated fatty acids (10.3 ± 5.9 g/day), with a total fat intake of 16.7 ± 6.0% of total energy intake. Therefore, it is possible that the protective effect of coffee consumption on MASLD development was attenuated by the well-balanced fat intake observed in this study. According to the Korea Agro-Fisheries & Food Trade Corporation [37], in the year 2000, coffee sales in Korea predominantly consisted of sugar-added coffee (156,700 tons), instant coffee (17,100 tons), and bean coffee (4000 tons). These data imply that the consumption of filtered coffee was relatively low. Consequently, the reduced lipid intake typically associated with filtered coffee might not have significantly influenced the results of our study.

The intake of carbohydrates, especially sugar, is also an important factor in the development of fatty liver. This study did not comprehensively evaluate sugar intake due to a lack of information; however, we indirectly assessed sugar consumption through the intake of sweetened beverages. Those who did not consume coffee also did not drink sweetened beverages. However, as coffee consumption increased, the intake of sweetened beverages remained similar across the groups, averaging about 0.3 cups/day. Therefore, the adverse effect of sugar intake on the development of MASLD may have remained consistent, potentially attenuating the protective effect of coffee consumption. Additionally, it is plausible that a substantial proportion of participants consumed additional sugar with their coffee because participants in the 2000s were most likely to drink sugar-added coffee. Despite the potential for added sugar intake, our study identified an association between coffee consumption and a reduced incidence of ALF. The protective effects of coffee consumption against ALF might be attenuated with the consumption of more than three cups, possibly due to the increased intake of additional sugar. It is possible that, had there been no added sugar intake, a more significant decrease in ALF incidence could have been observed with the consumption of more than three cups of coffee daily. Future research should assess the risk of ALF in relation to coffee intake while accurately accounting for added sugar intake.

Despite the highest proportion of current smokers and alcohol drinkers and the highest average total energy intake in the group with coffee consumption of ≥2 cups/day, the risk of ALF in this group was the lowest among the groups. Considering that there was no significant difference in serum hsCRP levels between the groups, this may partially demonstrate the anti-inflammatory effects of coffee consumption. Several potential mechanisms underlie the protective effects of coffee consumption against ALF. First, coffee consumption may downregulate profibrogenic genes, inhibit hepatic stellate cells adhesion and activation, and activate the Nrf2-induced antioxidant enzyme system, all of which contribute to the suppression of liver fibrosis [15]. Second, caffeine may inhibit adenosinergic signaling in liver myofibroblasts by acting on hepatic stellate cells A2aAR [16]. Caffeine prevented an increase in collagen levels and extracellular matrix deposition, attenuated fibrosis progression, and completely prevented increases in profibrogenic proteins TGF-β and α-smooth muscle actin in high-fat, high-sucrose, high-cholesterol diet-fed and thioacetamide-treated rats [38]. Finally, coffee intake may modulate the expression of long non-coding RNA involved in lipid metabolism, fibrogenesis, and circadian clock regulation. In mice fed a high-fat diet, coffee intake markedly decreases the hepatic expression of H19 and its target gene, collagen alpha-1(I) chain, which is involved in fibrosis. These findings suggest that coffee consumption contributes to the suppression of liver fibrosis by modulating the expression of long non-coding RNA [39] involved in lipid metabolism, fibrogenesis, and circadian clock regulation.

This study had several limitations. First, we defined MASLD and ALF using surrogate indices and not using more precise utilities such as computed tomography, magnetic resonance elastography, transient elastography, ultrasonography, and liver biopsy. Furthermore, we could not perform a sensitivity analysis using other surrogate markers to assess ALF such as the NAFLD fibrosis score or the Hepamet fibrosis score. Nevertheless, the NAFLD-liver fat score and FIB-4 score are well-validated indices for assessing MASLD and liver fibrosis, respectively, and have been used in many epidemiological studies. Second, we used only baseline information on lifestyle factors, including diet, coffee consumption, smoking status, drinking status, and physical activity. In particular, the dataset in the current study provided only baseline dietary information. As the amount of coffee consumed varies with time, we cannot guarantee that the dietary habits of each individual did not change during the follow-up period. Moreover, there may be a recall bias in the diet, which may be attributed to the imprecise assessment of the amount of coffee consumed. There was also a lack of information about whether the coffee was caffeinated/decaffeinated. However, to overcome this, a well-trained dietitian performed one-on-one interviews to assess each individual’s diet and minimize the recall bias. Additionally, given that decaffeinated coffee was not commonly consumed in Korea during 2001–2002, it is reasonable to assume that the majority of the coffee consumed during that period was caffeinated. Third, we could not verify genetic effects on the development of MASLD and ALF. Further genome-wide association studies that consider the interactions between genetic and environmental factors are needed to robustly examine the effects of coffee consumption on incident MASLD and ALF. Finally, our results may not be generalizable to other ethnicities because of the homogeneity of the study sample, which consisted of only Koreans. Differences in genetic risk factors and diet among different ethnic groups may limit the applicability of our findings to other populations. Despite these limitations, the strength of this study is that we verified the relationship between coffee consumption and incident ALF and MASLD in a large cohort of patients over a long-term follow-up period. We also suggest the most appropriate amount of coffee for the prevention of ALF in patients with MASLD.

## 5. Conclusions

Coffee consumption was associated with a reduced risk of incident ALF in patients with MASLD; however, it was not significantly associated with incident MASLD. The most appropriate amount of coffee consumed was between ≥2 and <3 cups per day. Our findings suggest that coffee consumption may serve as a cost-effective strategy to suppress the progression of liver fibrosis in patients with MASLD. Further studies considering genetic variations and environmental factors are needed to fully understand the relationship between coffee consumption and the progression of liver fibrosis.

## Figures and Tables

**Table 1:** Baseline characteristics of the study population.

	Individuals without MASLD					Individuals with MASLD and without ALF				
Variables	Without Coffee Consumption	>0 and <1 cups/day	≥1 and <2 cups/day	≥2 cups/day	*p* *	Without Coffee Consumption	>0 and <1 cups/day	≥1 and <2 cups/day	≥2 cups/day	*p* *
	(*n* = 1196)	(*n* = 1269)	(*n* = 1460)	(*n* = 1341)		(*n* = 330)	(*n* = 338)	(*n* = 351)	(*n* = 307)	
Male sex, *n* (%)	384 (32.1%)	527 (41.5%)	565 (38.7%)	710 (52.9%)	<0.001	95 (28.8%)	134 (39.6%)	158 (45.0%)	185 (60.3%)	<0.001
Age, years	54.7 ± 9.1	52.2 ± 8.8	50.7 ± 8.5	49.4 ± 8.0	<0.001	56.1 ± 8.0	54.5 ± 8.3	52.8 ± 8.4	50.8 ± 7.9	<0.001
BMI, kg/m^2^	23.5 ± 2.9	23.9 ± 2.8	24.1 ± 2.8	23.9 ± 2.9	<0.001	26.6 ± 2.9	26.7 ± 3.1	26.7 ± 2.9	26.8 ± 2.8	0.718
Mean blood pressure, mmHg	95.4 ± 12.9	94.7 ± 12.5	92.9 ± 12.5	92.8 ± 12.2	<0.001	104.3 ± 12.3	103.2 ± 12.6	102.2 ± 12.8	101.7 ± 11.1	0.034
Smoking status, *n* (%)					<0.001					<0.001
Never	918 (77.5%)	889 (70.8%)	976 (67.3%)	694 (52.3%)		258 (79.1%)	235 (69.9%)	222 (63.4%)	130 (42.6%)	
Former	138 (11.7%)	155 (12.3%)	185 (12.8%)	197 (14.8%)		37 (11.3%)	49 (14.6%)	56 (16.0%)	64 (21.0%)	
Intermittent	19 (1.6%)	35 (2.8%)	23 (1.6%)	33 (2.5%)		5 (1.5%)	6 (1.8%)	10 (2.9%)	6 (2.0%)	
Every day	109 (9.2%)	177 (14.1%)	266 (18.3%)	404 (30.4%)		26 (8.0%)	46 (13.7%)	62 (17.7%)	105 (34.4%)	
Current drinker, *n* (%)	343 (28.8%)	579 (45.8%)	686 (47.2%)	649(48.6%)	<0.001	89 (27.3%)	129 (38.3%)	152 (43.4%)	147 (47.9%)	<0.001
Physical activity, *n* (%)					<0.001					0.579
Low	93 (8.0%)	103 (8.5%)	83 (5.9%)	100 (7.7%)		27 (8.4%)	27 (8.4%)	26 (7.6%)	29 (9.7%)	
Moderate	636 (54.5%)	705 (57.9%)	958 (67.6%)	817 (62.6%)		180 (56.2%)	183 (56.8%)	214 (62.8%)	177 (59.4%)	
High	437 (37.5%)	409 (33.6%)	377 (26.6%)	389 (29.8%)		113 (35.3%)	112 (34.8%)	101 (29.6%)	92 (30.9%)	
Education, *n* (%)					<0.001					<0.001
Elementary/Middle school	816 (68.8%)	731 (58.0%)	713 (49.0%)	629 (47.2%)		250 (76.7%)	206(61.5%)	211 (60.3%)	151 (49.3%)	
High school	277 (23.4%)	361 (28.6%)	532 (36.6%)	494 (37.1%)		49 (15.0%)	92 (27.5%)	92 (26.3%)	102 (33.3%)	
College/University	93 (7.8%)	169 (13.4%)	210 (14.4%)	209 (15.7%)		27 (8.3%)	37 (11.0%)	47 (13.4%)	53 (17.3%)	
Monthly household income, KRW					<0.001					<0.001
<100 million	550 (46.9%)	459 (36.7%)	369 (25.6%)	365 (27.5%)		160 (50.0%)	150 (44.9%)	125 (35.9%)	92 (31.0%)	
100–200 million	338 (28.8%)	392 (31.4%)	466 (32.3%)	424 (31.9%)		82 (25.6%)	83 (24.9%)	85 (24.4%)	81 (27.3%)	
>200 million	285 (24.3%)	398 (31.9%)	607 (42.1%)	539 (40.6%)		78 (24.4%)	101 (30.2%)	138 (39.7%)	124 (41.8%)	
Platelet count, 10^9^/L	267.7 ± 63.6	263.2 ± 59.4	263.1 ± 59.5	268.4 ± 61.1	0.035	280.5 ± 60.4	270.5 ± 62.3	276.3 ± 63.5	280.5 ± 63.7	0.129
AST, U/L	26.7 ± 7.1	26.8 ± 7.4	26.3 ± 6.8	25.9 ± 6.4	0.003	32.3 ± 13.3	31.2 ± 12.0	33.0 ± 17.6	33.0 ± 18.2	0.385
ALT, U/L	21.5 ± 7.8	22.5 ± 8.7	22.1 ± 9.2	22.4 ± 9.5	0.013	36.2 ± 19.7	34.5 ± 19.2	38.9 ± 26.0	42.0 ± 38.1	0.002
r-GTP, U/L	22.3 ± 61.2	22.4 ± 24.3	22.1 ± 21.2	23.7 ± 23.4	0.667	37.1 ± 44.3	39.6 ± 49.2	46.5 ± 89.7	47.9 ± 60.2	0.085
Total bilirubin, mg/dL	0.6 ± 0.3	0.6 ± 0.3	0.6 ± 0.3	0.6 ± 0.3	0.005	0.5 ± 0.3	0.6 ± 0.3	0.6 ± 0.3	0.6 ± 0.3	0.085
Albumin, g/dL	4.2 ± 0.3	4.2 ± 0.3	4.3 ± 0.3	4.3 ± 0.3	<0.001	4.2 ± 0.3	4.2 ± 0.3	4.3 ± 0.3	4.3 ± 0.3	0.032
hsCRP, mg/dL	0.1 [0.0;0.2]	0.1 [0.0;0.2]	0.1 [0.1;0.2]	0.1 [0.0;0.2]	0.490	0.2 [0.1;0.3]	0.2 [0.1;0.3]	0.2 [0.1;0.3]	0.2 [0.1;0.3]	0.938
FPG, mg/dL	82.1 ± 10.3	82.7 ± 10.7	83.0 ± 14.5	82.2 ± 10.3	0.160	93.3 ± 26.7	92.9 ± 24.1	96.5 ± 28.8	96.9 ± 35.6	0.172
LDL cholesterol, mg/dL	113.2 ± 30.5	113.0 ± 29.0	117.7 ± 32.0	117.6 ± 30.4	<0.001	116.6 ± 32.0	116.8 ± 31.3	120.5 ± 30.6	123.3 ± 32.1	0.025
Total energy intake, kcal/day	2025.7 ± 807.2	2053.9 ± 783.2	2171.6 ± 703.4	2334.7 ± 921.4	<0.001	2035.8 ± 797.4	2100.0 ± 780.8	2186.2 ± 729.2	2460.2 ± 773.8	<0.001
CHO intake, % of total energy intake	69.0 ± 8.0	68.4 ± 7.8	66.7 ± 6.9	67.1 ± 7.0	<0.001	70.5 ± 7.3	69.7 ± 7.3	67.8 ± 7.4	67.3 ± 7.4	<0.001
Protein intake, % of total energy intake	13.6 ± 2.5	13.3 ± 2.3	13.5 ± 2.1	12.8 ± 2.2	<0.001	13.4 ± 2.4	13.3 ± 2.3	13.3 ± 2.2	12.9 ± 2.4	0.014
Fat intake, % of total energy intake	16.1 ± 6.4	17.0 ± 6.3	18.6 ± 5.6	18.9 ± 5.6	<0.001	14.9 ± 5.8	15.8 ± 5.8	17.6 ± 5.9	18.6 ± 5.9	<0.001
SFA intake, % of total energy intake	4.48 ± 1.98	4.30 ± 1.74	4.54 ± 1.70	4.21 ± 1.71	<0.001	4.19 ± 1.80	4.09 ± 1.57	4.30 ± 1.61	4.10 ± 1.57	0.325
MUFA intake, % of total energy intake	4.78 ± 2.17	4.61 ± 1.94	4.85 ± 1.85	4.56 ± 1.87	<0.001	4.53 ± 1.99	4.44 ± 1.79	4.63 ± 1.75	4.43 ± 1.65	0.439
PUFA intake, % of total energy intake	6.60 ± 2.73	5.93 ± 2.28	6.17 ± 2.31	5.80 ± 2.26	<0.001	6.52 ± 2.61	6.05 ± 2.40	6.18 ± 2.42	5.67 ± 2.29	<0.001
DM, *n* (%)	38 (3.2%)	40 (3.2%)	49 (3.4%)	24 (1.8%)	0.054	100 (30.3%)	89 (26.3%)	115 (32.8%)	78 (25.4%)	0.123
HTN, *n* (%)	406 (33.9%)	395 (31.1%)	402 (27.5%)	318 (23.7%)	<0.001	221 (67.0%)	216 (63.9%)	202 (57.5%)	181 (59.0%)	0.044
Dyslipidemia, *n* (%)	463 (38.7%)	476 (37.5%)	576 (39.5%)	521 (38.9%)	0.773	247 (74.8%)	241 (71.3%)	251 (71.5%)	236 (76.9%)	0.301
Coffee intake, cups/day	0.00 [0.00;0.00]	0.21 [0.11;0.50]	1.00 [1.00;1.00]	3.00 [2.00;3.00]	<0.001	0.00 [0.00;0.00]	0.21 [0.08;0.50]	1.00 [1.00;1.00]	3.00 [2.00;3.00]	<0.001
sweetened beverage intake, cups/day	0.00 [0.00;0.04]	0.03 [0.00;0.08]	0.03 [0.00;0.21]	0.03 [0.00;0.21]	<0.001	0.00 [0.00;0.03]	0.03 [0.00;0.08]	0.03 [0.00;0.21]	0.03 [0.00;0.21]	<0.001
NAFLD-liver fat score	−1.891 [−2.331;−1.344]	−1.900 [−2.311;−1.430]	−1.968 [−2.374;−1.440]	−1.922 [−2.341;−1.448]	0.106	0.151 [−0.284;0.817]	0.047 [−0.315;0.723]	0.183 [−0.319;0.864]	0.129 [−0.325;0.877]	0.659
FIB-4 score	1.17 [0.93;1.49]	1.11 [0.88;1.46]	1.08 [0.87;1.37]	1.01 [0.81;1.28]	<0.001	1.09 [0.85;1.34]	1.06 [0.85;1.44]	0.99 [0.80;1.27]	0.90 [0.72;1.22]	<0.001

* The *p*-value was calculated to compare the differences in continuous variables using analysis of variance and in categorical variables using the chi-square test among different coffee consumption groups. A *p*-value of less than 0.05 was considered statistically significant. Abbreviations: MASLD, metabolic dysfunction-associated steatotic liver disease; ALF, advanced liver fibrosis; BMI, body mass index; AST, aspartate aminotransferase; ALT, alanine aminotransferase; r-GTP, gamma-glutamyl transpeptidase; hsCRP, high-sensitivity C-reactive protein; FPG, fasting plasma glucose; LDL, low-density lipoprotein; CHO, carbohydrate; SFA, saturated fatty acids; MUFA, monounsaturated fatty acids; PUFA, polyunsaturated fatty acids; DM, diabetes mellitus; HTN, hypertension; NAFLD, nonalcoholic fatty liver disease; FIB-4, fibrosis-4.

**Table 2:** Cox proportional hazard regression analysis for the development of MASLD based on coffee consumption in participants without MASLD.

	Total Cases	New-Onset MASLD Cases	Person–Years	Incidence Rate per 1000 Person–Years	Unadjusted		Adjusted *	
Coffee consumption					HR (95% CI)	*p*	HR (95% CI)	*p*
Without coffee consumption	1196	504	12,267.6	41.08	1 (reference)		1 (reference)	
>0 and <1 cups/day	1269	564	13,117.3	43.00	1.04 (0.92–1.18)	0.497	1.04 (0.91–1.17)	0.595
≥1 and <2 cups/day	1460	664	15,030.2	44.18	1.07 (0.96–1.21)	0.231	1.05 (0.93–1.19)	0.447
≥2 cups/day	1341	566	13,832.6	40.92	1.00 (0.89–1.13)	0.970	0.98 (0.86–1.18)	0.769

* Adjusted for age, sex, BMI, physical activity, smoking status, alcohol drinking status, education level, monthly household income, total energy intake, average sweetened beverge consumption, whole blood platelet count, serum ALT, r-GTP, albumin, hsCRP levels, DM, HTN, and dyslipidemia. Abbreviations: MASLD, metabolic dysfunction-associated steatotic liver disease; HR, hazard ratio; CI, confidence interval; BMI, body mass index; ALT, alanine aminotransferase; r-GTP, gamma-glutamyl transpeptidase; hsCRP, high-sensitivity C-reactive protein; DM, diabetes mellitus; HTN, hypertension.

**Table 3:** Cox proportional hazard regression analysis for the development of ALF based on coffee consumption in patients with MASLD.

	Total Cases	New-Onset ALF Cases	Person–Years	Incidence Rate per 1000 Person–Years	Unadjusted		Adjusted *	
Coffee consumption					HR (95% CI)	*p*	HR (95% CI)	*p*
Without coffee consumption	330	70	4775.0	14.66	1 (reference)		1 (reference)	
>0 and <1 cups/day	338	81	4755.1	17.03	1.20 (0.87–1.65)	0.276	1.04 (0.73–1.49)	0.820
≥1 and <2 cups/day	351	57	5176.3	11.01	0.76 (0.53–1.07)	0.115	0.80 (0.55–1.17)	0.248
≥2 cups/day	307	42	4655.7	9.02	0.62 (0.43–0.91)	0.015	0.57 (0.37–0.90)	0.014

* Adjusted for age, sex, BMI, physical activity, smoking status, alcohol drinking status, education level, monthly household income, total energy intake, average sweetened beverge consumption, whole blood platelet count, serum ALT, r-GTP, albumin, hsCRP levels, DM, HTN, and dyslipidemia. Abbreviations: ALF, advanced liver fibrosis; MASLD, metabolic dysfunction-associated steatotic liver disease; HR, hazard ratio; CI, confidence interval; BMI, body mass index; ALT, alanine aminotransferase; r-GTP, gamma-glutamyl transpeptidase; hsCRP, high-sensitivity C-reactive protein; DM, diabetes mellitus; HTN, hypertension.

**Table 4:** Sensitivity analysis of coffee consumption and incidence of ALF in patients with MASLD, including 2–3 cups and 3 or more cups of coffee intake.

	Total Cases	New-Onset ALF Cases	Person–Years	Incidence Rate per 1000 Person–Years	Unadjusted		Adjusted *	
Coffee consumption					HR (95% CI)	*p*	HR (95% CI)	*p*
Without coffee consumption	330	70	4775.0	14.66	1 (reference)		1 (reference)	
>0 and <1 cups/day	338	81	4755.1	17.03	1.20 (0.87–1.65)	0.276	1.04 (0.73–1.49)	0.829
≥1 and <2 cups/day	351	57	5176.3	11.01	0.76 (0.53–1.07)	0.115	0.80 (0.55–1.17)	0.245
≥2 and <3 cups/day	141	19	2136.8	8.89	0.62 (0.37–1.02)	0.060	0.51 (0.30–0.89)	0.018
≥3 cups/day	166	23	2518.9	9.13	0.63 (0.39–1.09)	0.054	0.65 (0.38–1.12)	0.119

* Adjusted for age, sex, BMI, physical activity, smoking status, alcohol drinking status, education level, monthly household income, total energy intake, whole blood platelet count, serum ALT, r-GTP, albumin, hsCRP levels, DM, HTN, and dyslipidemia. Abbreviations: ALF, advanced liver fibrosis; MASLD, metabolic dysfunction-associated steatotic liver disease; HR, hazard ratio; CI, confidence interval; BMI, body mass index; ALT, alanine aminotransferase; r-GTP, gamma-glutamyl transpeptidase; hsCRP, high-sensitivity C-reactive protein; DM, diabetes mellitus; HTN, hypertension.

## Data Availability

The data used in this study were obtained from the Korean Genome and Epidemiology Study (KoGES; 4851-302), National Research Institute of Health, Korea Disease Control and Prevention Agency, Ministry for Health and Welfare, Republic of Korea. The dataset used in this study can be provided after a Korea Disease Control and Prevention Agency review and evaluation of the research plan (https://www.nih.go.kr/ko/main/contents.do?menuNo=300563 (accessed on 15 November 2023)).

## Supplementary Materials

The following supporting information can be downloaded at: https://www.mdpi.com/article/10.3390/nu16010140/s1; Figure S1: A Cox proportional hazard spline curve showing relation of the frequency of coffee intake per day with the risk of incident MASLD. Abbreviation: MASLD, metabolic dysfunction-associated steatotic liver disease; Figure S2: A Cox proportional hazard spline curve showing relation of the frequency of coffee intake per day with the risk of incident ALF in patients with MASLD. Abbreviations: ALF, advanced liver fibrosis; MASLD, metabolic dysfunction-associated steatotic liver disease.
