# Supplementary figures and images for: Different Associations of Coffee Consumption with the Risk of Incident Metabolic Dysfunction-Associated Steatotic Liver Disease and Advanced Liver Fibrosis
Source: Nutrients. 2023 Dec 31;16(1):140. doi: 10.3390/nu16010140 (PMC10781101; doi:10.3390/nu16010140)

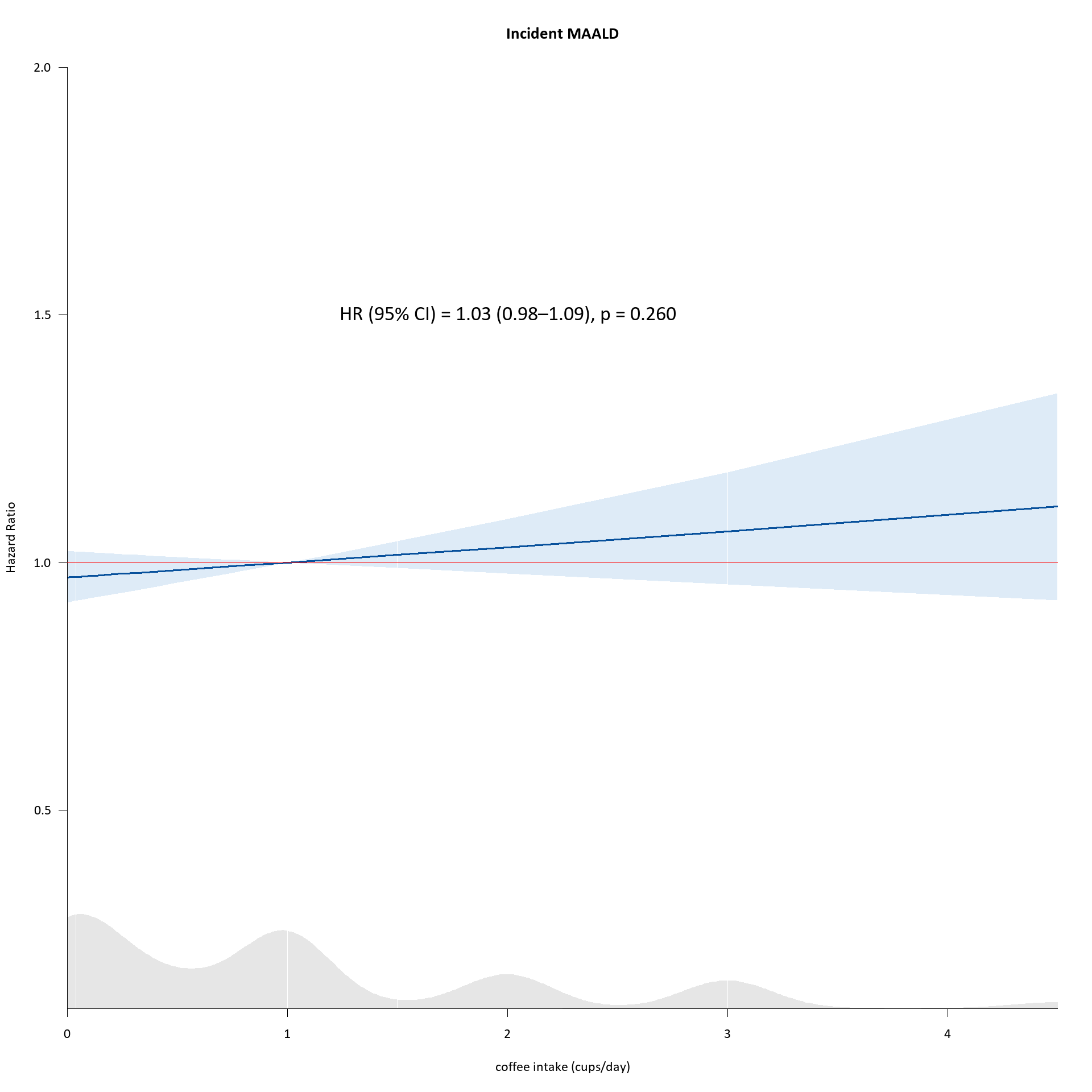

Supplement: Supplementary file 1 [file nutrients-16-00140-s001.zip › FigS1_1stRevision.tiff]

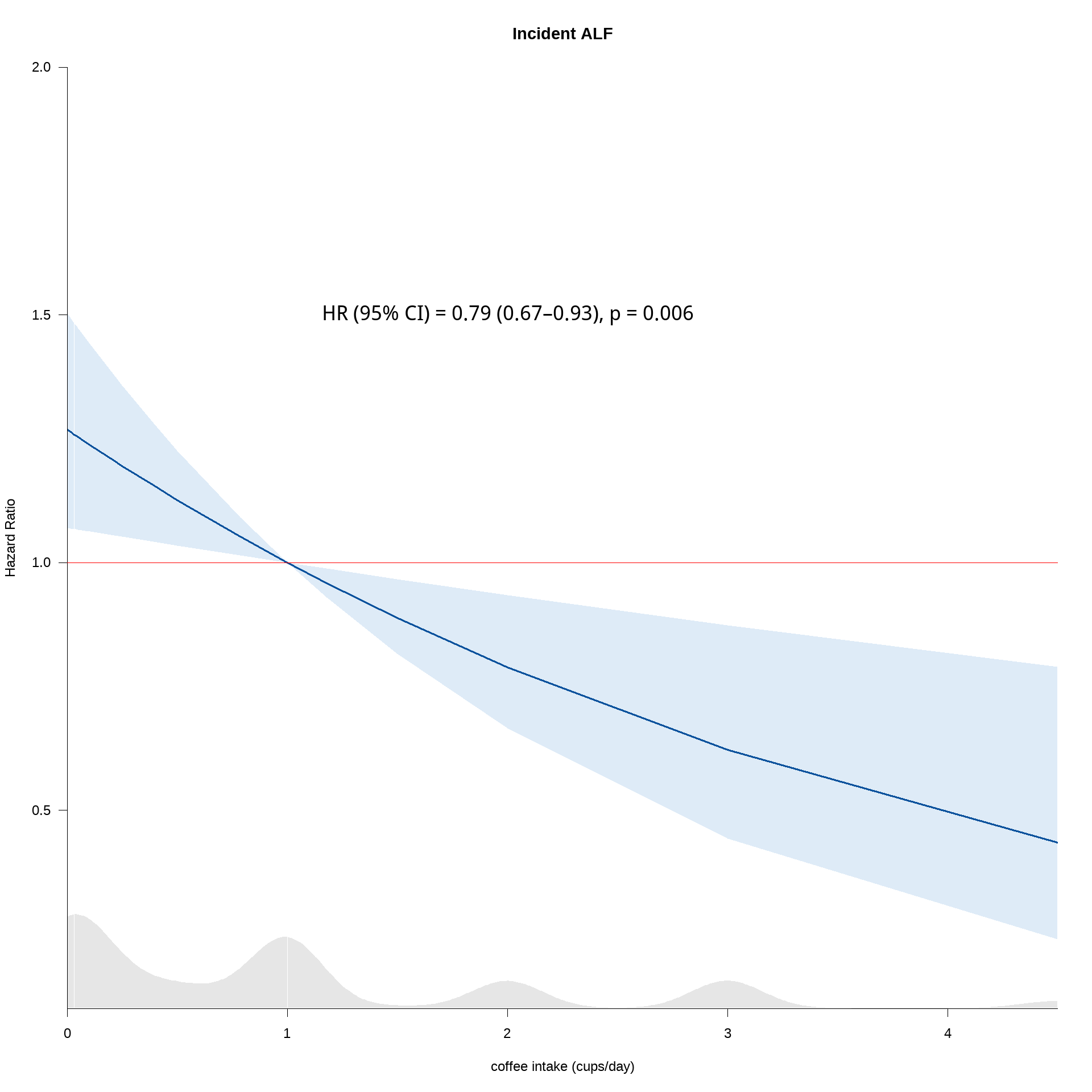

Supplement: Supplementary file 1 [file nutrients-16-00140-s001.zip › FigS2_1stRevision.tiff]
